# Abnormalities in red blood cell production and pathogenesis of anemia in the progression of rock bream iridovirus (RBIV)

**DOI:** 10.1016/j.virusres.2023.199278

**Published:** 2023-11-24

**Authors:** Soo-Jin Kim, Jayeon Cheon, Mi Young Cho, Sung-Ju Jung, Myung-Hwa Jung

**Affiliations:** a Pathology Division, National Institute of Fisheries Science, Republic of Korea; b Department of MarineBio and Medical Sciences, Hanseo University, Republic of Korea; c Department of Aqualife Medicine, Chonnam National University, Republic of Korea

**Keywords:** Rock bream, Rock bream iridovirus, Red blood cells, Anemia, Hematological indicators

## Abstract

Rock bream iridovirus (RBIV), belonging to *Megalocytivirus*, causes severe mortality in rock bream. Almost all deaths associated with RBIV are accompanied by splenic enlargement and anemia. Although red blood cells (RBCs) are involved in the immune response against viral infections, their involvement in rock bream has not yet been studied in terms of the immune response against RBIV. In this study, the viral replication patterns, blood characteristics and anemia-related factors were evaluated in rock bream post RBIV infection. The virus-infected RBCs of rock bream demonstrated similarities in the expression levels of hemoglobins (HGB) (α and β), cytokine-dependent hematopoietic cell linker (CLNK) and hematopoietic transcription factor GATA (GATA), with significantly decreasing levels from 4 days post infection (dpi) to 17 (dpi), when the viral replication was at its peak. This suggests that the expression of blood-related genes is inadequate for HGB synthesis and RBC production, thereby causing anemia leading to death. Moreover, the levels of complete blood cell count (CBC) indicators, such as RBCs, HGB and hematocrit (HCT), significantly decreased from 10 to 17 dpi. This phenomenon suggests that blood-related gene expression and/or RBC-, HGB- and HCT-related levels are critical factors in RBIV-induced anemia and disease progression. These results highlight the significance of blood-mediated immune responses against RBIV infection in rock bream. Understanding blood-related gene levels to identify blood-related immune response interactions in rock bream will be useful for development of future strategies in controlling RBIV diseases in rock bream.

## Highlights

• RBIV causes systemic infections in rock bream.
• Spleen reached its lethal level the fastest during RBIV infection.
• Hemoglobins (α and β), CLNK and GALT were lowest expressed in RBIV-infected rock bream.
• The RBC, HGB and HCT levels gradually decreased during RBV infection.

## 1 Introduction

The family *Iridoviridae* includes five genera: *Iridovirus, Chloriridovirus, Ranavirus, Lymphocystivirus* and *Megalocytivirus* (Williams et al., 2000). Rock bream iridovirus (RBIV), a member of the genus *Megalocytivirus* (Kurita and Nakajima, 2012), has been known to causes fatalities in rock bream (*Oplegnathus fasciatus*) in Korea (Jung and Oh, 2000). Our previous studies focused on fish mortality and pathogenicity, RBIV replication-dependent immune gene expression pattern, virus elimination, efficacy of immunostimulants, DNA vaccine experiments and formalin-inactivated vaccine experiments (Nikapitiya et al., 2014; Jung et al., 2014; Jung and Jung, 2017a; Jung and Jung, 2017b; Jung and Jung, 2017c; Jung and Jung, 2017d; Jung et al., 2022a; Jung et al., 2022b). However, the immune defense mechanisms of rock bream against RBIV remain unclear, considering that the virus is highly pathogenic to rock bream (Jung et al., 2015; Jung et al., 2016; Jung et al., 2018; Jung et al., 2019; Jung and Jung, 2019; Jung and Jung, 2021).

The spleen is the major site of pathogen growth and disease pathology in virus-affected rock bream (Jung and Oh, 2000) and is frequently used to diagnose damage caused by RBIV infections. The simplest method for diagnosing RBIV infection involves confirming the presence of abnormally enlarged cells on Giemsa-stained impression-smears of the spleen (Jung and Oh, 2000). Furthermore, when rock breams are infected, there is a marked increase in the weight of the spleen (up to approximately three-fold), indicating that spleen enlargement is positively correlated with RBIV replication (Jung et al., 2017a; Jung et al., 2017b; Jung and Jung, 2019). Although the genus *Megalocytivirus* comprises viruses associated with serious systemic infections that result in significant mortality, organs other than the spleen have not been commonly used to diagnose megalocytivirus infections. Moreover, although information regarding its etiology is limited, the clinical signs of the disease include anemia-related symptoms such as pale gills, increased respiration, lethargy, atypical swimming and negative buoyancy (Jung and Oh, 2000).

Massive deaths occur annually in Korean aquaculture fields owing to the combined effects of disease, low and high water temperature, typhoons, precipitation and red tides. Accordingly, minimizinge damage by inspecting fish health through regular surveillance is necessary; however, an established standard for determining these complex factors is lacking. Various indices have been used to evaluate the condition or well-being of fish, including the relative condition factor (Le Cren, 1951), gut index (Jensen, 1980), relative weight (Wege and Anderson, 1978), visceral somatic index (Adams et al., 1982; Delahunty and De Vlaming, 1980), RNA-DNA ratios of the liver and muscle (Bulow et al., 1981), and liver somatic index (Allen and Wootton, 1982; Bulow et al., 1978; Delahunty and De Vlaming, 1980; Edwards et al., 1972; Heidinger and Crawford, 1977; Tyler and Dunn, 1976; Valtonen, 1974). Them, liver somatic index is a useful biomarker for detecting environmental stressors, and is one of the most sensitive growth indicators (Allen and Wootton, 1982; Bulow et al., 1978; Edwards et al., 1972; Heidinger and Crawford, 1977; Tyler and Dunn, 1976; Valtonen, 1974). However, one disadvantage is that the fish must be sacrificed killed to test the indicators mentioned above. It is vital to obtain samples (e.g., blood) to measure the health of fish without killing them.

Therefore, various studies on blood cells, antibody titers, hemoglobin (HGB), hematocrit (HCT), pathogen-killing effects and immune responses can be conducted using the basic components of blood, white blood cells, red blood cells and plasma. However, the utility of these parameters in diagnosing and determining infections caused by pathogens is very low. This implies that barring a few pathogens that directly infect the blood, most pathogens target the spleen, kidneys, and heart as their primary target organs. There has been little interest in research using blood, where the proliferation of pathogens is relatively weak. Anemia may be useful among the different health measures that use blood. Anemia is not only caused specifically under a certain condition but also under multiple conditions, including water quality, dense diet, feed, environment and disease. Therefore, if the degree of anemia is quantified and compared to variations in a specific condition, and if a correlation is established between two, the condition may be used to assess health.

The aim of present study was to evaluate viral distribution in the organs of rock bream and the hematological changes following RBIV infection. Anemia was confirmed by measuring HCT, HGB and RBC levels according to the viral proliferation pattern, followed by a physiological analysis of blood. The expression levels of gene related to blood functions were also assessed in rock bream infected with RBIV.

## 2 Materials and methods

### 2.1 Experimental fish and virus

Rock breams free from RBIV were obtained from a local farm and reared at Hanseo University. The virus used in the present study was originally isolated from RBIV-infected rock bream in 2010 (RBIV-HD 2011, KT031401) (Jung et al., 2014). The major capsid protein (MCP) gene copies of RBIV in the supernatant preparations were quantified using quantitative real-time polymerase chain reaction (qPCR). The RBIV titer was calculated as 1.1 × 10^7^/100 μl MCP gene copies.

### 2.2 Variation in hematological parameters with RBIV infection

#### 2.2.1 Artificial infection

To determine mortality rate, 20 fish (10.4 ± 1.2 cm, 20.1 ± 2.4 g) in each group were divided randomly into two groups: the virus-injected group, which was intraperitoneally (i.p.) injected with RBIV (100 μl/fish) containing 10^6^ MCP gene copies, and the control group, which was administered phosphate-buffered saline (PBS). The fish were maintained at 23°C in an aquarium containing 20 L seawater.

Virus replication and hematological changes in rock bream were evaluated as follows: 60 fish (10.3 ± 0.9 cm, 20.3 ± 2.1 g) were maintained at 23°C in an aquarium. The fish were injected i.p. with RBIV (100 μl/fish, 10^6^ MCP gene copies) or PBS (100 μl/fish) as the control. The gill, liver, intestine, spleen, heart, kidney, muscle, brain and red blood cells (RBCs) were collected from five virus-injected fish and five control fish at 1, 4, 7, 10, 14, 16 and 17 days post infection (dpi). Blood (over 150 μl/fish) was collected at each sampling point; 50 μl of the blood samples was used for biochemical examination, 40 μl for hematocrit examination, 20 μl for the examination of complete blood cell count (CBC) and 40 μl was purified for RBCs. The RBCs were purified using two consecutive density gradient centrifugations (7206 *g*, Ficoll 1.007: Sigma Aldrich). The samples were stored at –80°C after being flash-frozen in liquid nitrogen.

#### 2.2.2 Examination of complete blood cell count (CBC)

Blood samples (20 μl/fish) were used to analyze CBC parameters, including hemoglobin (HGB), mean corpuscular hemoglobin (MCH), mean corpuscular hemoglobin concentration (MCHC), red blood cells (RBCs), mean corpuscular volume (MCV), hematocrit (HCT) and red blood cell distribution width (RDW) using an Exigo H400 (Boule Medical AB, Sweden). All analyses were performed according to the manufacturer's instruction.

Blood samples (40 μl/fish) were further used to analyze HCT. The HCT test measures the volume of RBCs compared to the total blood volume (red blood cells and plasma). The HCT tube was placed near the Eppendorf tube and blood was allowed to flow via capillary action into the HCT tube until two-thirds to three-fourths full or to a predesignated mark on the tube. The tubes were then placed in a microhematocrit centrifuge for 10 min at 10,000 rpm.

#### 2.2.3 Biochemical examination of blood

Blood samples (50 μl/fish) were used to analyze biochemical components, including total protein (TP), gamma-glutamyl peptidase (GGT), aspartate aminotransferase (AST/GOT), alanine aminotransferase (ALT/GPT), alkaline phosphatase (ALP), amylase (AMY), creatine (crea), blood urea nitrogen (BUN), Bun/Crea, total cholesterol (TC) and triglyceride (TG) using an Exigo C200 (Boule Medical AB, Sweden). Considering that the size of the fish was small, biochemical examination was performed using five volumes of blood and five volumes of PBS. All analyses were performed according to the manufacturer's instructions.

#### 2.2.4 Quantitative real-time PCR analysis of expression levels of blood-related genes

To analyze the expression of blood-related genes, total RNA was extracted from the kidney and RBCs using the RNAiso Plus reagent (TaKaRa, Japan), following the standard protocol. Total RNA was treated with DNase I (TaKaRa, Japan) and reverse-transcribed using the ReverTra Ace qPCR RT Kit (Toyobo, Japan) according to manufacturer's protocol. Real-time PCR was carried out in a CFX Connect Real-Time PCR Detection System (Bio-Rad, USA) using an AccuPre®2x Greenstar qPCR Master Mix (Bioneer, Korea) as described in a previous study (Jung et al., 2014). Each assay was performed in duplicate with β-actin RNA as the control. The primers used to amplify immune-related genes are listed in Table 1. The quantitation of the mRNA was determined using the 2^−ΔΔCt^ method (Livak and Schmittgen 2001).

**Table 1:** Primers used in this study.

Name	Sequence (Forward)	Sequence (Reverse)	Accession number
MCP	TGCACAATCTAGTTGAGGAGGTG	AGGCGTTCCAAAAGTCAAGG	AY849394
β-actin	CAGGGAGAAGATGACCCAGA	CATAGATGGGCACTGTGTGG	FJ975145
Hb-α	TATACCCGCAGACCAAGACC	TTTCCCACAGCTTCTCCAAC	MK681795.1
Hb-β	AGCGGTATTTCGGGACTTTT	CCTTTGTCCAGAGCCTTCAG	MK681796.1
CLNK	GTCCCGATGGAAAAGCCTCA	TTCACCAGGTGTAAGGCGTG	
GATA	TCTCCTCACCTACAGCCACA	CCAGTGTTGAGGTGGTGTGT	

### 2.3 Determination of viral copy numbers

Viral loads in the gill, liver, intestine, spleen, heart, kidney, muscle and RBCs samples (20 to 160 mg) were measured using qRT-PCR. Genomic DNA was isolated using the High Pure PCR Template Preparation Kit (Roche, Germany). The qRT-PCR procedure followed was 95°C for 10 min, 95°C for 15 s, and 60°C for 45 s for 40 cycles followed by scanning, as previously described (Jung et al., 2015). The details for establishing the standard curve have been previously described (Jung et al., 2015). The RBIV genome copy numbers were determined from 1 mg of organ weight of the gill, liver, intestine, spleen, heart, kidney, muscle and RBCs were quantified with specific MCP primer sets (Table 1).

### 2.4 Statistical analyses

Statistical analyses of gene expression and anemia related factor levels of rock bream were performed using unpaired t-tests using GraphPad Prism software version 5.0 for Windows (GraphPad Software, USA). Differences were considered statistically significant at p<0.001, p<0.05 and p<0.01. All the data are represented as the means ± the standard error.

## 3 Results

### 3.1 RBIV disease progression in rock bream

#### 3.1.1 Mortality rates

**Fig. 1:**
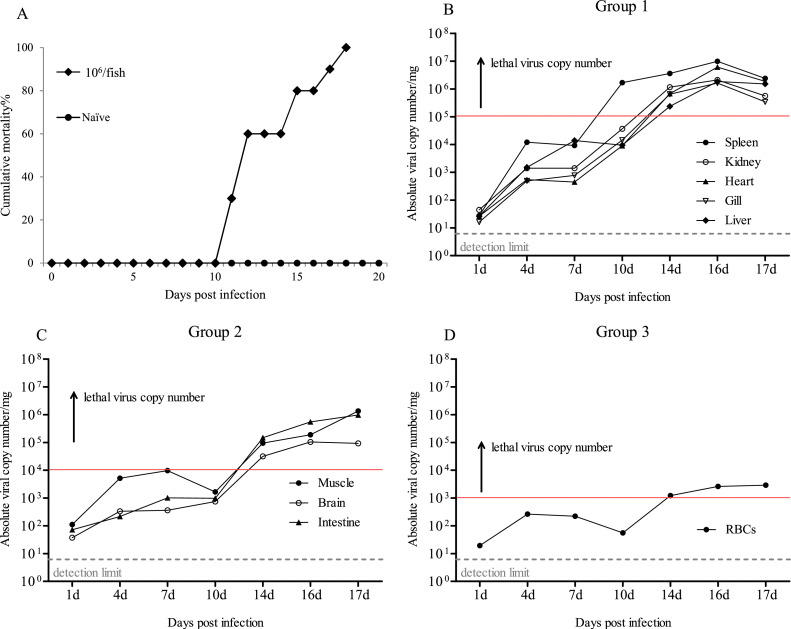
Rock breams were intraperitoneally (i.p.) injected with RBIV (10^6^ MCP gene copies/100 μl/fish) at 23°C. (A) Cumulative rock bream mortality. (B, C and D) Time-course replication analysis of three RBIV transcript classes. Viral replication profiles in different organs (gill, liver, intestine, spleen, heart, kidney, muscle and RBCs) replication profiles at 1, 4, 7, 10, 14, 16 and 17 days post-infection (dpi).

**Fig. 2:**
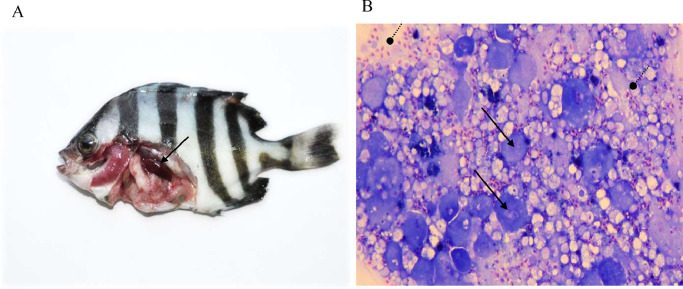
Dead rock bream exhibiting typical signs of RBIV infection. (A) enlarged spleen. (B) enlarged basophilic cells in the spleen imprint. Triangle and round arrows indicate enlarged basophilic and red blood cells, respectively.

In rock bream reared at 23°C, the mortality between 11 dpi and 18 dpi resulted in final mortality of 100 % (Fig. 1A). Dead rock bream exhibited typical signs of RBIV infection, such as enlarged basophilic cells in the spleen imprint and an enlarged spleen (Fig. 2).

#### 3.1.2 Three infection stages of RBIV

**Fig. 3:**
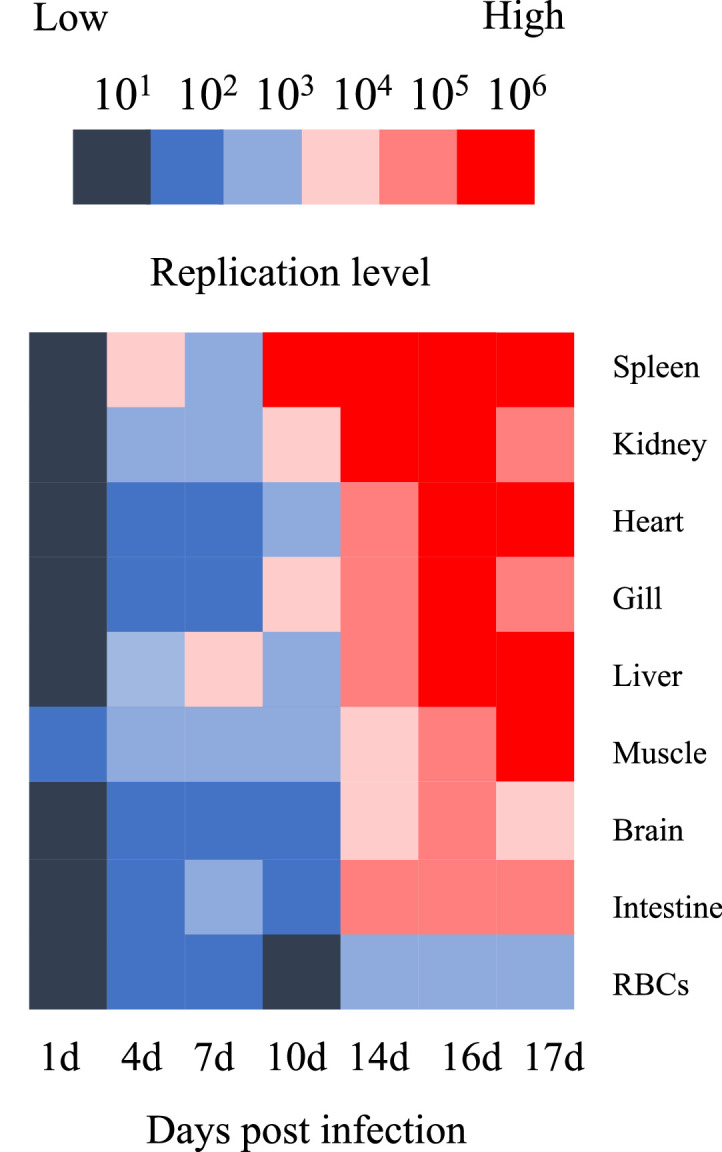
RBIV replication analysis of nine organs (gill, liver, intestine, spleen, heart, kidney, muscle and RBCs) at 1, 4, 7, 10, 14, 16 and 17 days post-infection (dpi). Red and blue boxes indicate high and low replication values, respectively.

The RBIV replication profiles were constructed and are presented in Fig. 1 and 3. Nine organs (gills, liver, intestine, spleen, heart, kidney, muscle, brain and RBCs) showed elevated viral replication following RBIV infection. Viral replication patterns were categorized into three infection stages: early (1 dpi), middle (4 to 10 dpi) and late (14 to 17 dpi) (Fig. 1 and 3).

At the early stage of infection at 1 dpi, increases (range: 10^1^–10^2^) in viral load were observed in all nine organs, and the infected fish appeared grossly healthy, demonstrating an increase in the number of infectious agents (Fig. 1).

The middle stage of the disease occurred between 4 and 10 dpi (viral load range: 10^1^–10^6^). This was followed by active replication of the virus, wherein the infected fish demonstrated clinical symptoms. Changes in the virus replication (range: 10^2^–10^3^) in the six organs (liver, muscle, brain, heart, gill and intestine) and RBCs showed elevated viral loads. However, the spleen and kidney showed high replication levels, with virus copy numbers of 1.70 × 10^6^ and 3.67 × 10^4^/mg, respectively (Supplementary Table 1) (Fig. 1).

The disease proceeded to the last stage between 14 and 17 dpi (range: 10^3^–10^6^). The viral load peaked exponentially and the fish demonstrated clinical symptoms with specific mortality. This period is regarded as the late stage of infection. Following initial RBIV infection, the number of replicated RBIV rapidly increased from 14 dpi. The RBIV load increased in all organs, indicating that intensive replication, transcription, and translation occurred to assemble and form viral progeny inside the infected host cells. At 16 dpi, the viral levels in the nine organs were significantly elevated. Among these organs, replication in six organs peaked at 16 dpi. In this study, the distribution of the virus based on the quantity in each organs was in the following order: spleen (average 9.92 × 10^6^), heart (6.14 × 10^6^), kidney (2.12 × 10^6^), liver (1.87 × 10^6^), gill (1.63 × 10^6^), intestine (5.53 × 10^5^), muscle (1.89 × 10^5^), brain (1.05 × 10^5^) and RBCs (2.62 × 10^3^). This indicated that most RBIV virions were completely assembled at 16 dpi (Fig. 1). At 17 dpi, viral level were elevated in nine organs were elevated, including the muscle, intestine and RBCs, which exhibited strongly replication (Fig. 1).

#### 3.1.3 Differential RBIV replication profiles

Specific mortality occurred between 11 and 18 dpi, and viral replication peaked between 14 and 17 dpi marking this period as the acute phase of infection (Fig. 1A and 3). Viral replication patterns at approximately 14 to 17 dpi, may be significant for evaluating the factor(s) involved in fish death. A lethal level was determined for each organ and all viral replication in the rock bream was clustered into three groups based on the similarity of replication patterns on 14 to 17 dpi as follows (Fig. 1 and 3): group 1, five organs (spleen, kidney, heart, gill and liver) showed high replication levels of >10^5^ at 14 dpi and this was regarded as the ‘lethal’ virus copy number (Fig. 1B); group 2, three organs (muscle, brain and intestine) exhibited high replication levels of >10^4^ at 14 dpi, which was regarded as the ‘lethal’ virus copy number (Fig. 1C); and group 3, which replication in one organ (RBCs) of >10^3^ at 14 dpi considered the ‘lethal’ virus copy number (Fig. 1D). The average viral load distribution in the fish organs was in the following order: >10^6^ (one organ: spleen), >10^5^ (six organs: heart, kidney, liver, intestine, gill and muscle), >10^4^ (one organ: brain) and >10^3^ (one organ: RBCs) (Fig. 1B, 1C and 1D).

### 3.2 Examination of complete blood cell count

#### 3.2.1 Anemia-related indicator levels

**Fig. 4:**
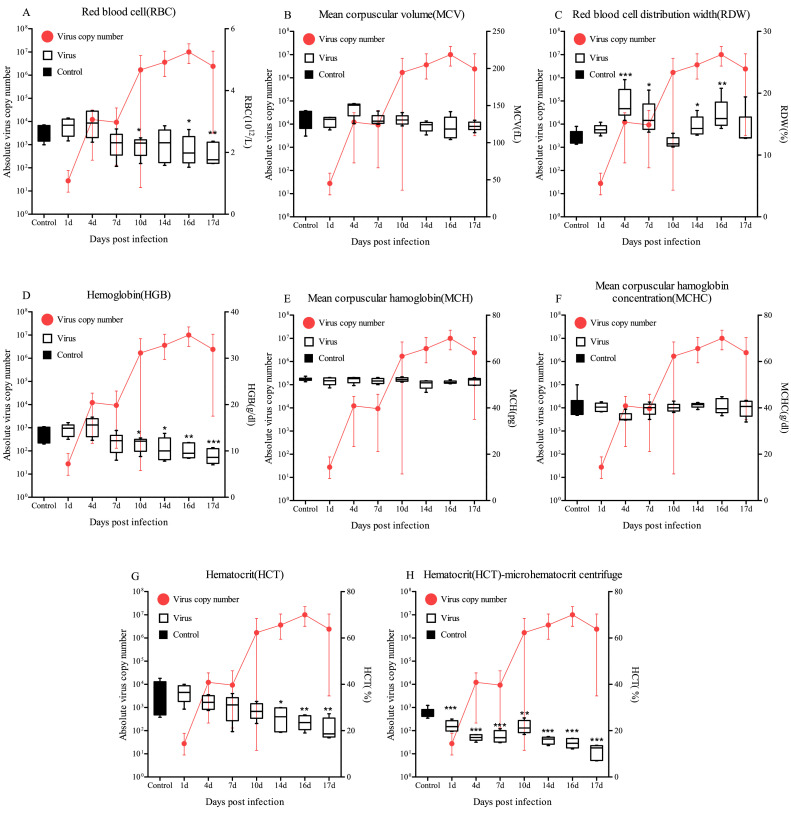
Complete blood cell count (CBC) examination of RBC, MCV, RDW, HGB, MCH, MCHC and HCT in the blood after injection with RBIV (10^6^ MCP gene copies/100 μl/fish) at 23°C. Bars indicate the standard error (SE) of the mean of five individuals; *p<0.05, **p<0.01 and ***p<0.001. The viral copy number in the spleen at each sampling point is plotted alongside for reference.

The RBC values in the control groups of PBS administered fish ranged between 2.25–2.89 (10^12^/L) and averaged 2.62 (10^12^/L) (Supplementary Table 2). The HGB values of the virus-infected group were lower than those of the PBS-injected fish, and gradually decreased from 7 dpi (average 2.26), 10 dpi (average 2.18) (*p*<0.01), 14 dpi (average 2.22), 16 dpi (average 2.06) (*p*<0.01) and 17 dpi (average 1.94) (*p*<0.05) (Fig. 4A).

The RDW values of control fish ranged from 11.74–13.80 ( %) with an average of 12.94 ( %) (Supplementary Table 2). The RDW values of RBIV-infected rock bream were lower than those of the control fish and gradually increased from 4 dpi (average 18.32) (*p*<0.001), 7 dpi (average 16.08) (*p*<0.01), 14 dpi (average 14.68) (*p*<0.01) and 16 dpi (average 16.52) (*p*<0.05) (Fig. 4C).

The HGB values of the control groups ranged between 11.4–15.2 (g/dl) and averaged 13.3 (g/dl) (Supplementary Table 2). The HGB values of RBIV-infected group were lower than those of the control group and gradually decreased from 7 dpi (average 11.6), 10 dpi (average 11.3) (*p*<0.01), 14 dpi (average 10.3) (*p*<0.01), 16 dpi (average 10.0) (*p*<0.05) and 17 dpi (average 8.8) (*p*<0.001) (Fig. 4D).

The HCT values of the control groups of the PBS administered fish ranged between 25.7–42.6 ( %), averaging 35.8 ( %) (Supplementary Table 2). The HCT values of RBIV-infected group were determined to be low compared with that of the control fish and gradually decreased from 7 dpi (average 29.6), 10 dpi (average 28.4), 14 dpi (average 25.2) (*p*<0.01), 16 dpi (average 23.4) (*p*<0.05) and 17 dpi (average 20.8) (*p*<0.05) (Fig. 3G). Based on the microhematocrit centrifugation results, the average HCT ranged between 21.0 to 30.9 % in the PBS-injected group (1 to 17 days post PBS injection). The RBIV-infected group, the average HCT levels significantly (*p*<0.001) decreased by 21.99, 17.06, 17.43, 21.63, 15.78, 14.55 and 10.76 % at 1, 4, 7, 10, 14, 16 and 17 dpi, respectively (Fig. 4H).

However, MCV, MCH, and MCHC values in the RBIV-infected group showed no significant differences compared to those in the control group (Fig. 4B, 4E, and 4F).

#### 3.2.2 Differential anemia-related indicator profiles

**Fig. 5:**
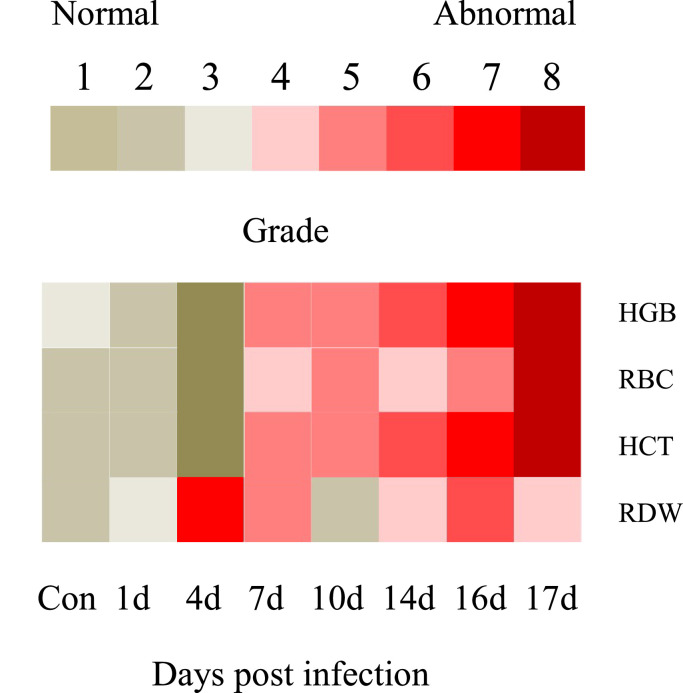
Classification of anemia severity in RBIV-infected rock bream. Red and yellowish-brown boxes indicate high and low grade values, respectively.

Grades of 1–8 were set for each anemia-related indicator in rock bream infected with RBIV (Table 2) (Fig. 5). A higher grade indicates a high-risk category for anemia owing to RBIV infections. Grade 1 for HGB, RBC, HCT, or RDW was set as 1 (g/dl), 0.2 (10^12^/L), 2.5 ( %) and 1 ( %), respectively. The anemia-related indicator grades for HGB, RBC, HCT, or RDW were as follows: i) at 14 dpi, 6, 4, 6 and 4, respectively; ii) at 16 dpi, 7, 5, 7 and 6, respectively; and iii) at 17 dpi, 8, 8, 8 and 4, respectively.

**Table 2:** Anemia severity classification for the RBIV-infected rock bream.

Description	No anemia	Mild anemia	Mild anemia	Mild anemia	Moderate anemia	Moderate anemia	Severe anemia	Very severe anemia
Grade	Level 1	Level 2	Level 3	Level 4	Level 5	Level 6	Level 7	Level 8
HGB (g/dl)	15∼16	15∼14	14∼13	13∼12	12∼11	11∼10	10∼9	9∼8
RBC (10^12^/L)	3.0∼2.8	2.8∼2.6	2.6∼2.4	2.4∼2.2	2.2∼2.0	2.0∼1.8	1.8∼1.6	1.6∼1.4
HCT ( %)	40∼37.5	37.5∼35	35∼32.5	32.5∼30	30∼27.5	27.5∼25	25∼22.5	22.5∼20
RDW ( %)	11∼12	12∼13	13∼14	14∼15	15∼16	16∼17	17∼18	18∼19

### 3.3 Biochemical examination of blood

#### 3.3.1 Biochemical indicator levels

**Fig. 6:**
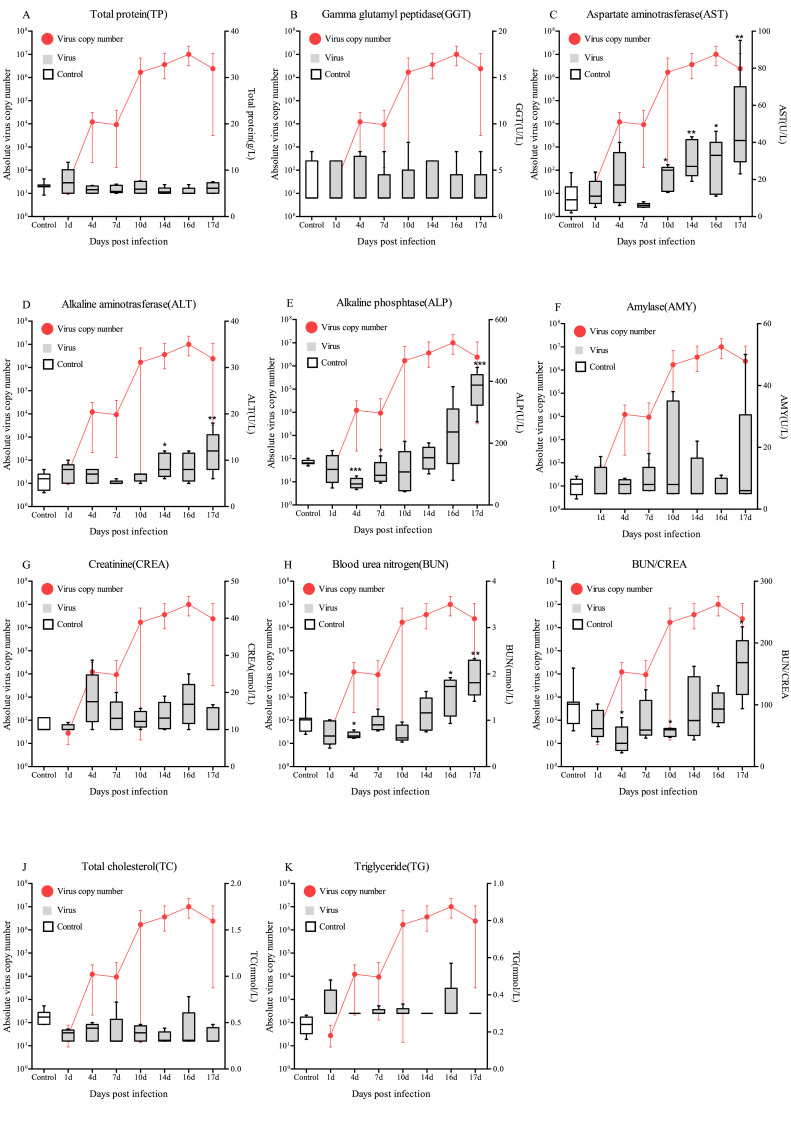
Biochemical examination of TP, GGT, AST, ALT, ALP, AMY, CREA, BUN, BUN/CREA, TC and TG in the blood after injection with RBIV (10^6^ MCP gene copies/100 μl/fish) at 23°C. Bars indicate the standard error (SE) of the mean of five individuals; *p<0.05, **p<0.01 and ***p<0.001. The viral copy number in the spleen at each sampling point is plotted alongside for reference.

The values for AST of the control fish ranged between 2.0–23.6 (U/L) with an average of 9.9 (U/L) (Supplementary Table 3). In the virus-infected fish, AST levels were higher than those of the control fish and significantly increased at 10 dpi (average 21.0) (*p*<0.01), 14 dpi (average 39.2) (*p*<0.05), 16 dpi (average 27.4) (*p*<0.01) and 17 dpi (average 67.2) (*p*<0.05) (Fig. 6C).

The ALT values of the control groups of the PBS administered fish ranged from 3.0–8.0 (U/L) and averaged 5.64 (U/L) (Supplementary Table 3). The ALP values of the virus-injected group were lower than those of the control fish and gradually increased from 14 (average 10.0) to 17 dpi (average 14.2) (*p*<0.001) (Fig. 6D).

The ALP values of the PBS administered fish ranged between 126.6–151.0 (U/L) and averaged 138.1 (U/L) (Supplementary Table 3). In RBIV-injected fish, ALP values were lower than those of the control fish and first gradually decreased from 1 dpi (average 86) to 10 dpi (average 108.8) and then gradually increased from 14 dpi (average 191.8) to 17 dpi (average 375.2) (*p*<0.001) (Fig. 6E).

The BUN values of the control groups of the PBS administered fish ranged between 0.7–1.5 (mmol/L) and averaged 1.0 (mmol/L) (Supplementary Table 3). In the virus-injected group, BUN values were lower than those of the control fish and gradually decreased from 1 dpi (average 0.74) to 10 dpi (average 0.71) followed by a gradual increase from 16 dpi (average 1.53) (*p*<0.01) to 17 dpi (average 1.90) (*p*<0.05) (Fig. 6H).

However, no differences were observed in the levels of TP, GGT, AMY, CREA, TC and TG in virus-injected fish compared to those in the control (Fig. 6A, 6B, 6D, 6F, 6G, 6J and 6K).

#### 3.3.2 Differential biochemical indicators profiles

**Fig. 7:**
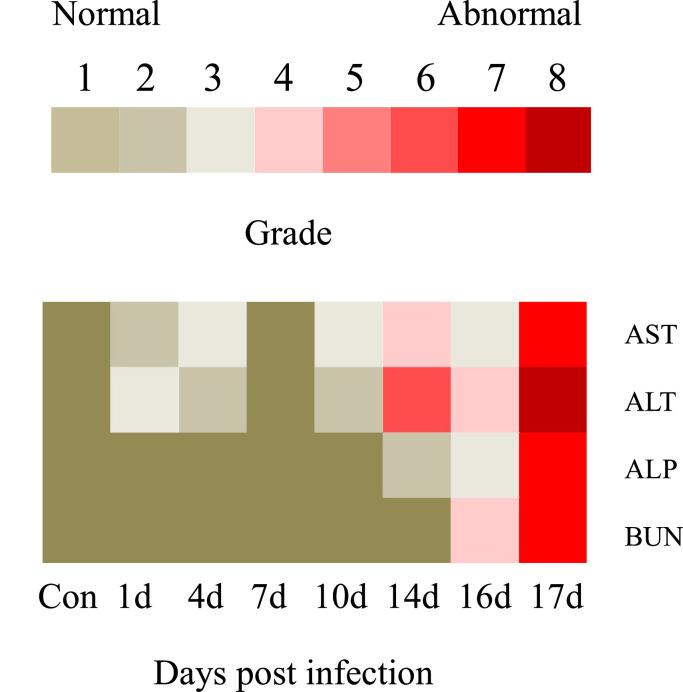
Classification of hamotological parameter severity in RBIV-infected rock bream. Red and yellowish-brown boxes indicate high and low grade values, respectively.

Grades 1 to 8 were established for each biochemical-related indicator in the rock bream under RBIV infection (Table 3) (Fig. 7). A higher grade correlated with a high risk for RBIV infection. Grade 1 for AST, ALT, ALP and BUN were defined as 10 (U/L), 1 (U/L), 40 (U/L) and 0.15 (μmol/L), respectively. The biochemical indicator grades for AST, ALT, ALP and BUN were as follows: i) at 14 dpi, 4, 5, 2 and 1, respectively, ii) at 16 dpi, 3, 4, 3 and 4, respectively and iii) at 17 dpi, 7, 8, 7 and 7, respectively).

**Table 3:** Hematological parameter classification for the RBIV-infected rock bream.

Description	Low replication	Mild replication	Mild replication	Mild replication	Moderate replication	Moderate replication	Severe replication	Very severe replication
Grade	Level 1	Level 2	Level 3	Level 4	Level 5	Level 6	Level 7	Level 8
AST (U/L)	0∼10	10∼20	20∼30	30∼40	40∼50	50∼60	60∼70	70∼80
ALT (U/L)	5∼6	6∼7	7∼8	8∼9	9∼10	10∼11	11∼12	12∼13
ALP (U/L)	120∼160	160∼200	200∼240	240∼280	280∼320	320∼360	360∼400	400∼440
BUN (umol/L)	1.00∼1.15	1.15∼1.30	1.30∼1.45	1.45∼1.60	1.60∼1.75	1.75∼1.90	1.90∼2.05	2.05∼2.20

### 3.4 Blood-related immune gene expression

**Fig. 8:**
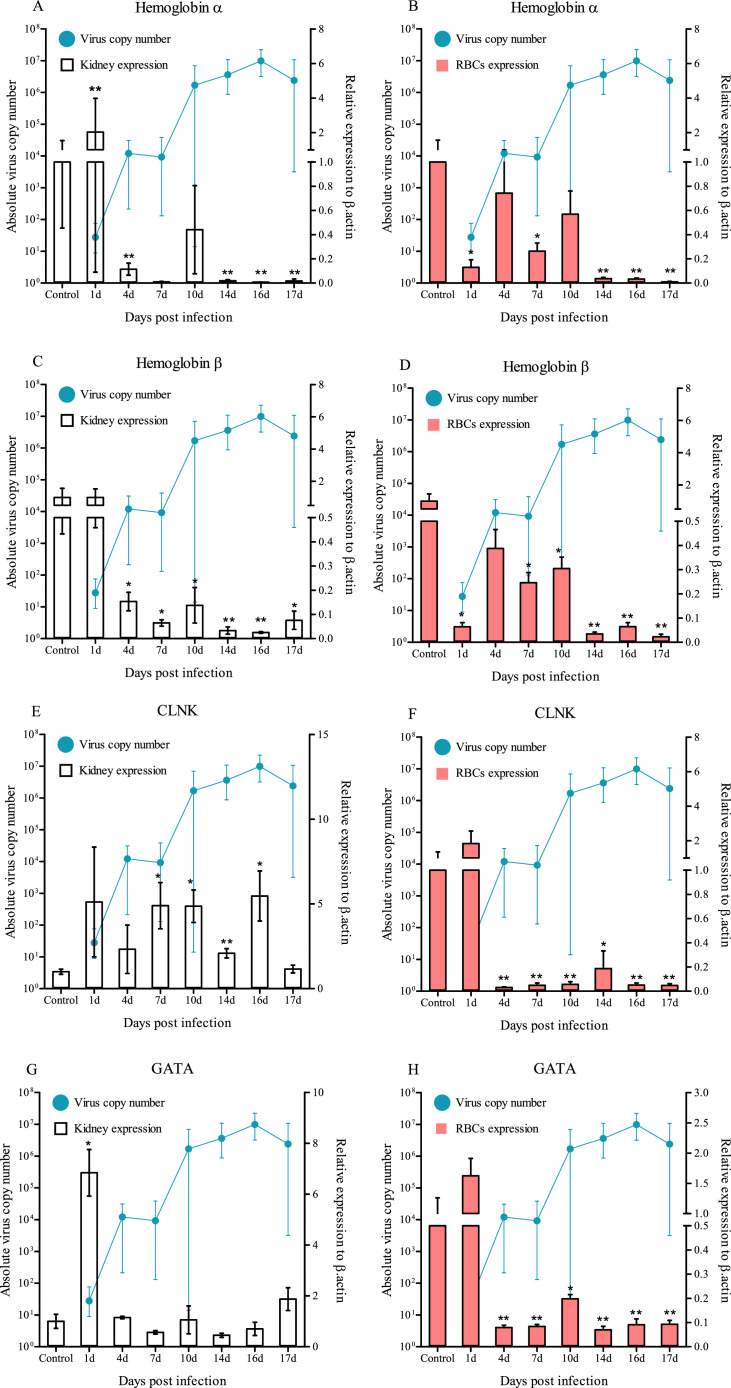
Relative expressions levels of blood-related genes in the RBCs and kidney at 1, 4, 7, 10, 14, 16 and 17 days post-infection (dpi). Expression levels were normalized against β-actin used as an internal control for a real-time PCR-based relative expression study. Bars indicate the standard error (SE) of the mean of five individuals; *p<0.05, **p<0.01. A-Hb-α, B-HB-β, C-CLNK and D-GATA. The viral copy number in the spleen at each sampling point is plotted alongside for reference.

Similar trends in gene expression were observed for hemoglobin α and hemoglobin β between the kidney and RBCs in the artificial infection experiment (Fig. 8A, 8B, 8C and 8D). In the kidney, differences in hemoglobin α mRNA expression were observed at 1 dpi (2.04-fold), 4 dpi (0.11-fold) (*p*<0.05), 7 dpi (0.01-fold) (*p*<0.05), 10 dpi (0.44-fold), 14 dpi (0.02-fold) (*p*<0.05), 16 dpi (0.01-fold) (*p*<0.05) and 17 dpi (0.02-fold) (*p*<0.05) in the RBIV-infected fish compared with control fish (Fig. 8A). Differing hemoglobin β mRNA expression was observed at 1 dpi (1.00-fold), 4 dpi (0.15-fold) (*p*<0.01), 7 dpi (0.06-fold) (*p*<0.01), 10 dpi (1.27-fold) (*p*<0.01), 14 dpi (0.03-fold) (*p*<0.05), 16 dpi (0.03-fold) (*p*<0.05) and 17 dpi (0.08-fold) (*p*<0.01) in the RBIV-infected fish compared with that in the control fish (Fig. 8C).

Significantly higher expression levels of cytokine-dependent hematopoietic cell linker (CLNK) were observed in the kidney at 7 dpi (4.8-fold) (*p*<0.01), 10 dpi (4.8-fold) (*p*<0.01), 14 dpi (2.8-fold) (*p*<0.05) and 16 dpi (5.4-fold) (*p*<0.01) than in the control group (Fig. 8E). However, a significant decrease in CLNK expression was observed from 4 to 17 dpi in the RBCs (Fig. 8F).

The expression of hematopoietic transcription factor GATA (GATA) decreased further from 1 dpi (6.8-fold) (*p*<0.01) to below the basal level (4 to 17 dpi) in the kidney (Fig. 8G). However, GATA expression was significantly decreased by less than 0.3-fold (4 to 17 dpi) in the RBCs (Fig. 8H) (*p*<0.01) (*p*<0.05).

## 4 Discussion

*Megalocytivirus* includes viruses associated with serious systemic infections that can cause significant mortality. Organs, other than the spleen have not been commonly studied to evaluate megalocytivirus infections. The present study revealed similarities in RBIV replication patterns in nine organs (spleen, liver, muscle, brain, gill, kidney, heart, intestine and RBCs). The acute stage of RBIV infection was observed to occur from 4 to 10 dpi in all organs of the RBIV-infected rock bream, with viral replication reached its peak at 16 to 17 dpi. The RBIV copy numbers were not as high as those in the RBCs and other organs. Nonetheless, time-dependent increses were observed in the blood and Ficoll-purified RBCs of rock bream indicating that RBIV causes systemic infections in rock bream.

The patterns of viral copy number and spleen size in virus-infected rock bream have been evaluated previously to determine the lethal and safe levels of the viral copy number/or the spleen index, which may define disease progression (Jung et al., 2017b; Jung and Jung, 2019). In these previous studies, upon dead (100 % mortality, at 20–29°C), the virus copy number and spleen index (spleen weight / fish weight × 100) were 10^8^–10^10^/100 μl and 3.00–5.38, respectively. Conversely, at survival (0 % mortality, at 17°C), the virus copy number and spleen index were as low as those of the not-infected control (10^2^–10^3^/100 μl and 0.34–1.22, respectively). In the present study, a threshold (dangerous limit) was determined for the viral replication pattern in the nine organs including mortality points indicating a possible viral copy number, that leads to fish death.

Mortality was observed between 11 and 18 dpi and viral replication peaked in all organs between 14 and 17 dpi. Lethal levels were set for each organ at 14 and 17 dpi. The lethal level for each organ was in the order of: 10^5^/mg (spleen, kidney, heart, gill and liver), 10^4^/mg (muscle, brain and intestine) and 10^3^/mg (RBCs). The organs were organized according to the lethal levels in the following order: spleen, kidney, heart, liver, gill, intestine, muscle, brain and RBCs. Among them, the lethal level was achieved the quickest in the spleen, confirming its preference for the diagnosis of RBIV infection.

Red blood cells (RBCs) play a key physiological role in the transport of oxygen from the lungs to tissues via hemoglobin. In mammals, mature RBCs lack nuclei, organelles and ribosomes (Moras et al., 2017). In contrast, with a few exceptions, non-mammalian RBCs (particularly in fish), nucleate and contain organelles in their cytoplasm (Nombela and Ortega-Villaizan, 2018). Nucleated RBCs offer numerous advantages over non-nucleated RBCs, and teleost RBCs can induce toll-like receptors (TLRs), phagocytosis by macrophages and cytokines or interferons (Dahle et al., 2015; Nombela et al., 2017; Nombela and Ortega-Villaizan, 2018; Passantino et al., 2007; Rodriguez et al., 2005). This indicates that in addition to their unique function in oxygen transport, the red blood cells of fish contribute to the host immune response to infectious diseases. Our previous study uncovered clues about the interaction between RBIV infection and the red blood cells or blood of rock bream: i) The rock bream administered with poly (I:C) demonstrated significantly elevated expression levels of interferon regulatory factor 3 (IRF3), interferon-stimulated gene 15 (ISG15) and protein kinase RNA-activated (PKR) genes in the blood; however, there was no significant up-regulation observed in the hematopoietic organ (spleen and kidney) (Jung and Jung 2017d), ii) Furthermore, the highest mRNA expression levels for the MHC class I gene were in the entire blood of the rock bream and was the lowest in the kidney and brain (Nikapitiya et al., 2014), iii) In RBIV-infected individuals, a total of 318 proteins were significantly regulated in the RBCs; 183 proteins were up-regulated and 135 proteins were down-regulated. These findings indicate that RBCs in rock bream infected with RBIV may induce a response to the infection. This response is characterized by the induction of apoptosis, MHC class I, and spliceosome pathways and downregulation of ISG15 antiviral mechanisms (Jung et al., 2019). Thus, RBCs may have a critical immune defense mechanism in rock bream. Massive deaths, occur if RBCs are unable to recover their function because of viral infections.

Whether blood function-related gene alterations occur owing to RBIV infection was investigated in the present study. The mRNA expression levels of the subunits of hemoglobin (Hb) protein, Hb-α and Hb-β in the RBCs, gradually decreased below 1-fold from 4 to 17 dpi. CLNK is an adaptor protein that regulates immune receptor signaling and signal transduction by interacting with various signaling proteins (Hakeim et al., 2021). The mRNA expression of CLNK increased approximately 2-fold at 1 dpi and which decreased after 4 dpi. Additionally, the mRNA expression of GATA, which plays a role in erythropoiesis (Fujiwara 2017), increased after 1 dpi and then decreased after 4 dpi. These results indicate that blood-related genes are unreactive in the early stages of RBIV infection, suggesting that RBC production is insufficient to activate immune response signals, especially hemoglobin synthesis, which is suppressed in RBIV-infected rock bream. Of note, the highest and lowest mRNA expression levels of CLNK and GATA were observed in the kidneys and RBCs, respectively. This indicates that various blood-related immune responses occur between hematopoietic organs (kidneys) and RBCs during RBIV infection. These findings may improve the current understanding of RBIV and host interactions and aid in the clarification of RBIV pathogenesis.

Analysis of blood parameters plays a key role in determining the health of an organism and its physiological status. Blood sampling and analyses are routinely performed in human, veterinary and physiological studies. Anemia in fish is the lack of oxygen-transporting red blood cells in the body (Hoffbrand et al., 2001) and has been described in several teleost fish (Witeska, 2015). The importance and relevance of investigating anemia can be explained as follows: i) The most apparent symptom of anemia in a fish is pale gills, resulting from a lack of red blood cell flow, ii) Anemia is an abnormal condition where the body lacks red blood cells, making it incapable of adequately transporting oxygen, iii) In blood tests, this appears as low hemoglobin concentration, the primary protein in red blood cells, iv) Low red blood cell counts may be observed when the body's HGB does not function; it can not produce enough hemoglobin when it breaks down RBCs too rapidly or lacks RBCs, vi) The symptoms of anemia, arise considering essential organs do not receive enough oxygen to function correctly. Thus, anemia is frequently used to diagnose the damage caused by pathogenic infections.

Studies on fish diseases such as *Renibacterium salmoninarum* (Delghandi et al., 2020), vibrio species (Austin, 2006), infectious hematopoietic necrosis (IHN) (St-Hilaire et al., 2002), viral hemorrhagic septicemia (VHS) (Raja-Halli et al., 2006; Stone et al., 2008; Dale et al., 2009), erythrocytic inclusion body syndrome (EIBS) in salmonids (Rodger et al., 1991; Jarp et al., 1996; Rodger and Richards, 1998) and viral erythrocytic necrosis (VEN) (Rohovec and Amandi, 1981) have been reported. Moreover, HCT values between 25 % and 50 % have been recorded in rock bream, olive flounder (*Paralichthys olivaceus*) and the atlantic salmon (*Salmo salar*), with some individuals demonstrating an HCT of <10 % (Park et al., 2017; Park et al., 2017; Currie et al., 2022). One of the few consistent characteristics of clinical anemia in salmon farms is the rapid decline in HCT values and the onset of mortality following an initial outbreak (Currie et al., 2022). The types of anemia may be classified based on changes in RBC volume (microcytic: smaller (iron deficiency anemia, thalassemia and sideroblastic anemia); normocytic: normal (anemia of chronic disease and aplastic anemia); macrocytic: larger (megaloblastic anemia and hemolytic anemia)), Hb concentrations (hypochromic: reduced; normochromic: normal), loss of blood cells (hemolytic or hemorrhagic) and hemopoietic activity (regenerative and nonregenerative) (Tvedten, 2010). *Megalocytivirus* can cause aplastic anemia and contribute to death (Kawato et al., 2017). To the best of our knowledge, no published information is available to assess the nature of this recent health challenge, and no current hematological reference values are available for normally farmed rock bream or *Megalocytivirus*.

The most apparent symptom of anemia in RBIV-infected rock bream is pale gills, which may result from a lack of red blood cell flow. This was evident from the anemia-related indicators that showed a gradual increase in virus copy numbers (approximately 10^5^/mg) which was accompanied by a gradual decrease in HGB, RBC and HCT levels between 14 and 17 dpi. This indicated that anemia was induced, followed by a decrease in RBC count, HGB concentration and HCT level, ultimately leading to death. These findings may indicate that the hemoglobin ((<10 (g/dl)), RBC ((<2.0 (10^12^/L)) and HCT ((<20 ( %)) results in severe anemia when virus replication reached its peak.

In summary, this study demonstrated that blood-related indicators such as hemoglobin (α and β), CLNK and GATA may not be activated in RBIV infection and that their immune responses are critical factors for RBIV anemia. The results of this study provide a list of new hematological targets that may be used to further explore RBIV disease mechanisms. The role of blood or RBCs remains unclear, indicating that the absence of studies focusing on rock bream blood severely hampers elucidation of the pathogenic mechanisms underlying RBIV infection. More detailed studies are, required to obtain a better understanding of the morphological changes in blood cells (white blood cells, lymphocytes, monocytes, granulocytes, thrombocytes and RBCs) and the functions related to immune cell defense interactions in rock bream during RBIV infection.

## CRediT authorship contribution statement

**Soo-Jin Kim:** Data curation, Writing – original draft. **Jayeon Cheon:** Writing – review & editing. **Mi Young Cho:** Writing – review & editing. **Sung-Ju Jung:** Writing – review & editing. **Myung-Hwa Jung:** Data curation, Writing – original draft, Investigation.

## Declaration of Competing Interest

The authors declare that they have no known competing financial interests or personal relationships that could have appeared to influence the work reported in this paper.

## Data Availability

I have shared my data.

## Appendix Supplementary materials

Image, application 1
