# Supplementary material for: Abnormalities in red blood cell production and pathogenesis of anemia in the progression of rock bream iridovirus (RBIV)
Source: Virus Res. 2023 Nov 24;339:199278. doi: 10.1016/j.virusres.2023.199278 (PMC10709172; doi:10.1016/j.virusres.2023.199278)
Supplement: Supplementary file 1 [file mmc1.docx]

**Supplementary Table 1**. Summary of virus copy number in the collected fish’s organs after RBIV infection.

| Days | Liver | Muscle | Brain | Spleen | Kidney | Heart | Gill | Intestine | Red blood cells | Average |
| --- | --- | --- | --- | --- | --- | --- | --- | --- | --- | --- |
| 1 day | 2.64 × 10^1^ | 1.11 × 10^2^ | 3.73 × 10^1^ | 2.77 × 10^1^ | 4.48 × 10^1^ | 2.64 × 10^1^ | 1.61 × 10^1^ | 7.22 × 10^1^ | 1.94 × 10^1^ | 4.24 × 10^1^ |
| 4 days | 1.51 × 10^3^ | 5.17 × 10^3^ | 3.33 × 10^2^ | 1.21 × 10^4^ | 1.41 × 10^3^ | 5.43 × 10^2^ | 4.89 × 10^2^ | 2.17 × 10^2^ | 2.64 × 10^2^ | 2.45 × 10^3^ |
| 7 days | 1.39 × 10^4^ | 9.70 × 10^3^ | 3.64 × 10^2^ | 9.27 × 10^3^ | 1.41 × 10^3^ | 4.45 × 10^2^ | 7.78 × 10^2^ | 1.01 × 10^3^ | 2.22 × 10^2^ | 4.12 × 10^3^ |
| 10 days | 9.51 × 10^3^ | 1.65 × 10^3^ | 7.52 × 10^2^ | 1.70 × 10^6^ | 3.67 × 10^4^ | 9.07 × 10^3^ | 1.44 × 10^4^ | 9.77 × 10^2^ | 5.58 × 10^1^ | 1.97 × 10^5^ |
| 14 days | 2.38 ×10^5^ | 9.53 × 10^4^ | 3.17 × 10^4^ | 3.63 × 10^6^ | 1.16 × 10^6^ | 6.76 × 10^5^ | 6.49 × 10^5^ | 1.48 × 10^5^ | 1.23 × 10^3^ | 7.36 × 10^5^ |
| 16 days | 1.87 × 10^6^ | 1.89 × 10^5^ | 1.05 × 10^5^ | 9.92 × 10^6^ | 2.12 × 10^6^ | 6.14 × 10^6^ | 1.63 × 10^6^ | 5.52 × 10^5^ | 2.62 × 10^3^ | 2.50 × 10^6^ |
| 17 days | 1.53 × 10^6^ | 1.36 × 10^6^ | 9.30 × 10^4^ | 2.41 × 10^6^ | 5.63 × 10^5^ | 1.89 × 10^6^ | 3.43 × 10^5^ | 9.73 × 10^5^ | 2.90 × 10^3^ | 1.02 × 10^6^ |
| Average | 5.22 × 10^5^ | 2.38 × 10^5^ | 3.30 × 10^4^ | 2.52 × 10^6^ | 5.55 × 10^5^ | 1.25 × 10^6^ | 3.77 × 10^5^ | 2.39 × 10^5^ | 1.04 × 10^3^ |  |

**Supplementary Table 2**. Complete blood cell count examination for the RBIV-infected rock bream at 23 °C.

| Group | | HGB (g/dl) | MCH (pg) | MCHC (g/dl) | RBC (10^12^/L) | MCV (fl) | RDW (%) | HCT (%)* | HCT (%)** |
| --- | --- | --- | --- | --- | --- | --- | --- | --- | --- |
| Control | 1 day | 11.70 | 51.87 | 49.97 | 2.36 | 108.82 | 11.74 | 25.76 | 25.40 |
|  | 4 days | 11.48 | 51.26 | 43.14 | 2.25 | 118.98 | 12.22 | 26.74 | 26.10 |
|  | 7 days | 15.10 | 52.64 | 36.78 | 2.87 | 143.08 | 13.08 | 41.02 | 29.10 |
|  | 10 days | 15.24 | 52.78 | 37.12 | 2.89 | 142.20 | 11.90 | 41.12 | 30.90 |
|  | 14 days | 12.92 | 51.96 | 37.30 | 2.49 | 139.40 | 14.64 | 34.76 | 27.30 |
|  | 16 days | 13.40 | 52.13 | 38.65 | 2.84 | 142.10 | 13.20 | 42.60 | 27.60 |
|  | 17 days | 13.80 | 53.62 | 40.96 | 2.65 | 135.60 | 13.80 | 38.60 | 26.80 |
|  | Average | 13.38 | 52.32 | 40.56 | 2.62 | 132.88 | 12.94 | 35.80 | 27.60 |
| Virus  1 day | 1 | 12.50 | 52.70 | 42.60 | 2.37 | 123.70 | 14.00 | 29.30 | 20.00 |
|  | 2 | 16.10 | 53.10 | 40.30 | 3.04 | 131.50 | 15.30 | 40.00 | 19.61 |
|  | 3 | 14.90 | 51.70 | 38.50 | 2.88 | 134.00 | 14.20 | 38.70 | 23.58 |
|  | 4 | 15.10 | 48.60 | 41.50 | 3.11 | 117.20 | 13.10 | 36.50 | 21.74 |
|  | 5 | 13.70 | 51.30 | 38.30 | 2.67 | 133.90 | 14.10 | 35.80 | 25.00 |
|  | Average | 14.46 | 51.48 | 40.24 | 2.81 | 128.06 | 14.14 | 36.06 | 21.99 |
| Virus  4 days | 1 | 13.80 | 52.60 | 34.90 | 2.62 | 150.60 | 19.00 | 39.60 | 15.00 |
|  | 2 | 16.60 | 49.60 | 39.40 | 3.35 | 125.70 | 15.60 | 42.20 | 16.67 |
|  | 3 | 12.30 | 53.00 | 34.70 | 2.33 | 152.60 | 22.20 | 35.50 | 18.29 |
|  | 4 | 15.60 | 53.10 | 35.00 | 2.95 | 151.50 | 17.30 | 44.70 | 18.29 |
|  | 5 | 17.30 | 52.00 | 35.50 | 3.32 | 146.60 | 17.50 | 48.70 | 17.05 |
|  | Average | 15.12 | 52.06 | 35.90 | 2.91 | 145.40 | 18.32 | 42.14 | 17.06 |
| Virus  7 days | 1 | 14.40 | 52.20 | 40.10 | 2.76 | 130.10 | 16.00 | 36.00 | 14.83 |
|  | 2 | 11.30 | 50.00 | 35.00 | 2.27 | 142.60 | 20.50 | 32.50 | 20.83 |
|  | 3 | 12.20 | 50.90 | 39.40 | 2.41 | 129.00 | 15.60 | 31.10 | 15.31 |
|  | 4 | 8.00 | 51.60 | 40.60 | 1.55 | 127.00 | 14.60 | 19.60 | 19.23 |
|  | 5 | 12.30 | 53.10 | 42.50 | 2.31 | 124.90 | 13.70 | 28.90 | 16.95 |
|  | Average | 11.64 | 51.56 | 39.52 | 2.26 | 130.72 | 16.08 | 29.62 | 17.43 |

| Group | | HGB (g/dl) | MCH (pg) | MCHC (g/dl) | RBC (10^12^/L) | MCV (fl) | RDW (%) | HCT (%)* | HCT (%)** |
| --- | --- | --- | --- | --- | --- | --- | --- | --- | --- |
| Virus 10 days | 1 | 8.80 | 53.40 | 38.00 | 1.64 | 140.50 | 13.50 | 23.10 | 19.86 |
|  | 2 | 12.80 | 51.80 | 39.20 | 2.47 | 131.90 | 12.10 | 32.60 | 21.15 |
|  | 3 | 12.20 | 52.20 | 40.00 | 2.34 | 130.60 | 11.30 | 30.60 | 18.37 |
|  | 4 | 12.10 | 52.60 | 42.90 | 2.30 | 122.60 | 11.60 | 28.30 | 23.26 |
|  | 5 | 11.00 | 51.10 | 40.10 | 2.16 | 127.50 | 11.80 | 27.50 | 25.49 |
|  | Average | 11.38 | 52.22 | 40.04 | 2.18 | 130.62 | 12.06 | 28.42 | 21.63 |
| Virus 14 days | 1 | 8.40 | 46.70 | 42.30 | 1.58 | 110.50 | 14.30 | 19.90 | 17.41 |
|  | 2 | 11.80 | 51.70 | 41.30 | 1.75 | 125.20 | 13.60 | 28.50 | 13.57 |
|  | 3 | 10.00 | 50.80 | 39.30 | 2.59 | 129.30 | 17.20 | 25.60 | 16.9 |
|  | 4 | 7.80 | 51.40 | 41.40 | 2.86 | 124.20 | 15.10 | 18.80 | 16.38 |
|  | 5 | 13.80 | 50.20 | 41.40 | 2.31 | 121.10 | 13.20 | 33.30 | 14.65 |
|  | Average | 10.36 | 50.16 | 41.14 | 2.22 | 122.06 | 14.68 | 25.22 | 15.78 |
| Virus 16 days | 1 | 8.40 | 50.80 | 43.20 | 1.80 | 118.40 | 14.30 | 26.10 | 14.55 |
|  | 2 | 11.70 | 51.00 | 39.20 | 2.28 | 104.20 | 15.90 | 19.00 | 12.86 |
|  | 3 | 9.50 | 50.40 | 36.60 | 1.98 | 141.80 | 20.80 | 26.80 | 12.13 |
|  | 4 | 11.80 | 52.10 | 39.60 | 1.52 | 108.80 | 16.30 | 23.50 | 16.67 |
|  | 5 | 8.60 | 50.90 | 44.80 | 2.74 | 126.60 | 15.30 | 21.80 | 16.52 |
|  | Average | 10.00 | 51.04 | 40.68 | 2.06 | 119.96 | 16.52 | 23.44 | 14.55 |
| Virus 17 days | 1 | 7.60 | 52.30 | 40.60 | 1.64 | 130.10 | 12.70 | 18.60 | 13.64 |
|  | 2 | 7.00 | 53.00 | 33.90 | 2.30 | 113.50 | 12.90 | 16.90 | 12.57 |
|  | 3 | 8.60 | 52.10 | 42.50 | 1.76 | 125.10 | 12.80 | 17.50 | 13.45 |
|  | 4 | 10.30 | 49.80 | 38.80 | 2.36 | 122.00 | 12.80 | 27.30 | 7.02 |
|  | 5 | 10.70 | 49.70 | 43.20 | 1.65 | 121.60 | 19.40 | 23.60 | 7.14 |
|  | Average | 8.84 | 51.38 | 39.80 | 1.94 | 122.46 | 14.12 | 20.78 | 10.76 |

*Complete blood cell count (CBC) used

**Microhemacrocrit centrifuge used

**Supplementary Table 3**. Biochemical examination of blood for the RBIV-infected rock bream at 23 °C.

| Group | | ALB  (g/L) | TP  (g/L) | TB  (umol/L) | GGT  (U/L) | AST  (U/L) | ALT  (U/L) | ALP  (U/L) | AMY  (U/L) | CREA  (umol/L) | UA  (umol/L) | BUN  (umol/L) | BUN/CREA | TC  (umol/L) | TG  (umol/L) |
| --- | --- | --- | --- | --- | --- | --- | --- | --- | --- | --- | --- | --- | --- | --- | --- |
| Control | 1 day | 6.40 | 6.47 | 1.00 | 6.00 | 16.00 | 7.00 | 126.67 | 10.67 | 10.00 | 10.00 | 0.70 | 69.67 | 0.49 | 0.28 |
|  | 4 days | 5.50 | 6.80 | 1.60 | 4.00 | 23.67 | 8.00 | 144.33 | 3.33 | 10.00 | 10.00 | 1.04 | 104.33 | 0.48 | 0.16 |
|  | 7 days | 5.40 | 4.63 | 1.00 | 7.00 | 9.67 | 3.50 | 136.00 | 6.67 | 10.00 | 10.00 | 1.59 | 159.33 | 0.68 | 0.20 |
|  | 10 days | 6.00 | 8.13 | 1.00 | 2.00 | 2.00 | 3.00 | 133.67 | 9.67 | 13.20 | 10.00 | 0.76 | 57.83 | 0.48 | 0.19 |
|  | 14 days | 5.30 | 6.50 | 1.00 | 2.00 | 3.33 | 5.00 | 151.00 | 4.67 | 13.20 | 10.00 | 1.00 | 76.01 | 0.58 | 0.25 |
|  | 16 days | 5.60 | 6.40 | 1.00 | 2.00 | 6.00 | 6.00 | 140.00 | 8.10 | 10.00 | 10.00 | 1.01 | 101.00 | 0.56 | 0.24 |
|  | 17 days | 5.80 | 6.80 | 1.00 | 2.00 | 9.00 | 7.00 | 135.00 | 8.60 | 10.00 | 10.00 | 1.03 | 103.00 | 0.61 | 0.29 |
|  | Average | 5.71 | 6.53 | 1.09 | 3.57 | 9.95 | 5.64 | 138.10 | 7.39 | 10.91 | 10.00 | 1.02 | 95.88 | 0.56 | 0.23 |
| Virus  1 day | 1 | 5.00 | 7.30 | 1.00 | 6.00 | 5.00 | 5.00 | 91.00 | 17.00 | 10.70 | 10.00 | 0.66 | 61.35 | 0.30 | 0.30 |
|  | 2 | 5.00 | 5.00 | 1.00 | 2.00 | 9.00 | 8.00 | 55.00 | 5.00 | 10.00 | 10.00 | 0.40 | 40.00 | 0.41 | 0.30 |
|  | 3 | 5.00 | 8.50 | 1.00 | 6.00 | 24.00 | 8.00 | 176.00 | 10.00 | 11.90 | 10.00 | 0.96 | 80.52 | 0.39 | 0.37 |
|  | 4 | 5.00 | 5.00 | 1.00 | 2.00 | 11.00 | 10.00 | 115.00 | 5.00 | 10.00 | 10.00 | 0.57 | 57.00 | 0.30 | 0.30 |
|  | 5 | 5.00 | 11.70 | 1.00 | 2.00 | 14.00 | 5.00 | 143.00 | - | 10.00 | 10.00 | 1.10 | 110.00 | 0.43 | 0.48 |
|  | Average | 5.00 | 7.50 | 1.00 | 3.60 | 12.60 | 7.20 | 115.00 | 9.25 | 10.52 | 10.00 | 0.74 | 69.77 | 0.37 | 0.35 |
| Virus  4 days | 1 | 5.00 | 6.70 | 1.00 | 2.00 | 29.00 | 8.00 | 78.00 | 5.00 | 10.00 | 21.00 | 0.79 | 79.00 | 0.30 | 0.30 |
|  | 2 | 5.00 | 5.80 | 1.00 | 2.00 | 17.00 | 7.00 | 94.00 | 5.00 | 17.50 | 10.00 | 0.66 | 37.75 | 0.44 | 0.30 |
|  | 3 | 5.00 | 5.00 | 1.00 | 6.00 | 6.00 | 5.00 | 50.00 | 5.00 | 20.70 | 10.00 | 0.61 | 29.57 | 0.50 | 0.30 |
|  | 4 | 5.00 | 5.00 | 1.00 | 2.00 | 9.00 | 5.00 | 61.00 | 5.00 | 14.20 | 10.00 | 0.70 | 48.95 | 0.30 | 0.30 |
|  | 5 | 5.00 | 6.50 | 1.00 | 7.00 | 40.00 | 8.00 | 68.00 | 5.00 | 28.70 | 10.00 | 0.64 | 22.38 | 0.46 | 0.30 |
|  | Average | 5.00 | 5.80 | 1.00 | 3.80 | 20.20 | 6.60 | 70.20 | 5.00 | 18.22 | 12.20 | 0.68 | 43.53 | 0.40 | 0.30 |
| Virus  7 days | 1 | 5.00 | 5.00 | 1.00 | 2.00 | 8.00 | 5.00 | 71.00 | 6.00 | 20.00 | 10.00 | 0.92 | 46.10 | 0.30 | 0.30 |
|  | 2 | 5.00 | 7.00 | 1.00 | 2.00 | 5.00 | 6.00 | 159.00 | 8.00 | 10.00 | 10.00 | 1.24 | 124.00 | 0.35 | 0.34 |
|  | 3 | 5.00 | 5.30 | 1.00 | 2.00 | 6.00 | 5.00 | 82.00 | 9.00 | 13.00 | 10.00 | 0.77 | 59.17 | 0.30 | 0.30 |
|  | 4 | 5.00 | 5.40 | 1.00 | 7.00 | 5.00 | 5.00 | 96.00 | 6.00 | 14.80 | 10.00 | 0.82 | 55.57 | 0.30 | 0.30 |
|  | 5 | 5.00 | 6.50 | 1.00 | 2.00 | 6.00 | 5.00 | 115.00 | 18.00 | 10.00 | 10.00 | 0.90 | 90.00 | 0.72 | 0.30 |
|  | Average | 5.00 | 5.84 | 1.00 | 3.00 | 6.00 | 5.20 | 104.60 | 9.40 | 13.56 | 10.00 | 0.93 | 74.97 | 0.39 | 0.31 |

| Group | | ALB  (g/L) | TP  (g/L) | TB  (umol/L) | GGT  (U/L) | AST  (U/L) | ALT  (U/L) | ALP  (U/L) | AMY  (U/L) | CREA  (umol/L) | UA  (umol/L) | BUN  (umol/L) | BUN/CREA | TC  (umol/L) | TG  (umol/L) |
| --- | --- | --- | --- | --- | --- | --- | --- | --- | --- | --- | --- | --- | --- | --- | --- |
| Virus 10 days | 1 | 5.00 | 7.70 | 1.00 | 2.00 | 25.00 | 6.00 | 139.00 | 38.00 | 14.00 | 10.00 | 0.83 | 59.61 | 0.39 | 0.35 |
|  | 2 | 5.00 | 5.90 | 1.00 | 2.00 | 25.00 | 7.00 | 43.00 | 5.00 | 10.00 | 10.00 | 0.62 | 62.00 | 0.30 | 0.30 |
|  | 3 | 5.00 | 7.70 | 1.00 | 8.00 | 13.00 | 5.00 | 107.00 | 8.00 | 11.30 | 10.00 | 0.53 | 47.31 | 0.48 | 0.30 |
|  | 4 | 5.00 | 5.00 | 1.00 | 2.00 | 14.00 | 7.00 | 49.00 | 5.00 | 12.20 | 10.00 | 0.61 | 50.06 | 0.30 | 0.30 |
|  | 5 | 5.00 | 5.00 | 1.00 | 2.00 | 28.00 | 7.00 | 206.00 | 32.00 | 15.70 | 10.00 | 0.96 | 61.32 | 0.45 | 0.30 |
|  | Average | 5.00 | 6.26 | 1.00 | 3.20 | 21.00 | 6.40 | 108.80 | 17.60 | 12.64 | 10.00 | 0.71 | 56.06 | 0.38 | 0.31 |
| Virus 14 days | 1 | 5.00 | 6.90 | 1.00 | 6.00 | 27.00 | 8.00 | 132.00 | 5.00 | 10.40 | 10.00 | 1.34 | 128.58 | 0.30 | 0.30 |
|  | 2 | 5.00 | 5.00 | 1.00 | 2.00 | 85.00 | 17.00 | 401.00 | 22.00 | 19.00 | 10.00 | 0.82 | 43.24 | 0.36 | 0.30 |
|  | 3 | 5.00 | 5.40 | 1.00 | 2.00 | 19.00 | 6.00 | 101.00 | 11.00 | 15.60 | 10.00 | 1.16 | 74.55 | 0.31 | 0.30 |
|  | 4 | 5.00 | 5.30 | 1.00 | 2.00 | 40.00 | 12.00 | 153.00 | 5.00 | 10.00 | 10.00 | 1.62 | 162.00 | 0.44 | 0.33 |
|  | 5 | 5.00 | 5.00 | 1.00 | 6.00 | 25.00 | 7.00 | 172.00 | 5.00 | 13.10 | 10.00 | 0.75 | 57.23 | 0.30 | 0.30 |
|  | Average | 5.00 | 5.52 | 1.00 | 3.60 | 39.20 | 10.00 | 191.80 | 9.60 | 13.62 | 10.00 | 1.14 | 93.12 | 0.34 | 0.31 |
| Virus 16 days | 1 | 5.00 | 5.00 | 1.00 | 2.00 | 13.00 | 6.00 | 383.00 | 9.00 | 10.00 | 10.00 | 0.93 | 93.00 | 0.78 | 0.30 |
|  | 2 | 5.00 | 5.00 | 1.00 | 2.00 | 11.00 | 5.00 | 80.00 | 5.00 | 19.30 | 10.00 | 1.25 | 64.82 | 0.30 | 0.30 |
|  | 3 | 5.00 | 5.00 | 1.00 | 2.00 | 34.00 | 11.00 | 236.00 | 5.00 | 16.80 | 10.00 | 1.81 | 107.51 | 0.31 | 0.30 |
|  | 4 | 5.00 | 6.90 | 1.00 | 7.00 | 46.00 | 12.00 | 238.00 | 11.00 | 25.00 | 10.00 | 1.92 | 76.85 | 0.43 | 0.30 |
|  | 5 | 5.00 | 5.30 | 1.00 | 2.00 | 33.00 | 8.00 | 188.00 | 5.00 | 13.20 | 10.00 | 1.73 | 130.97 | 0.30 | 0.57 |
|  | Average | 5.00 | 5.44 | 1.00 | 3.00 | 27.40 | 8.40 | 225.00 | 7.00 | 16.86 | 10.00 | 1.53 | 94.63 | 0.42 | 0.35 |
| Virus 17 days | 1 | 5.00 | 5.00 | 1.00 | 7.00 | 36.00 | 10.00 | 269.00 | 5.00 | 15.10 | 10.00 | 1.41 | 93.80 | 0.30 | 0.30 |
|  | 2 | 5.00 | 5.00 | 1.00 | 2.00 | 23.00 | 6.00 | 376.00 | 5.00 | 10.00 | 10.00 | 2.26 | 226.00 | 0.30 | 0.30 |
|  | 3 | 5.00 | 7.50 | 1.00 | 2.00 | 191.00 | 30.00 | 388.00 | 105.00 | 16.70 | 10.00 | 2.33 | 139.41 | 0.48 | 0.30 |
|  | 4 | 5.00 | 7.10 | 1.00 | 2.00 | 45.00 | 12.00 | 398.00 | 11.00 | 10.00 | 10.00 | 1.68 | 168.00 | 0.41 | 0.30 |
|  | 5 | 5.00 | 6.10 | 1.00 | 2.00 | 41.00 | 13.00 | 445.00 | 6.00 | 10.00 | 10.00 | 1.81 | 181.00 | 0.30 | 0.30 |
|  | Average | 5.00 | 6.14 | 1.00 | 3.00 | 67.20 | 14.20 | 375.20 | 26.40 | 12.36 | 10.00 | 1.90 | 161.64 | 0.36 | 0.30 |
